# Soluble PD-L1: A biomarker to predict progression of autologous transplantation in patients with multiple myeloma

**DOI:** 10.18632/oncotarget.11519

**Published:** 2016-08-23

**Authors:** Shang-Yi Huang, Hsiu-Hsia Lin, Chung-Wu Lin, Chi-Cheng Li, Ming Yao, Jih-Luh Tang, Hsin-An Hou, Woei Tsay, Sheng-Je Chou, Chieh-Lung Cheng, Hwei-Fang Tien

**Affiliations:** ^1^ Department of Internal Medicine, National Taiwan University, Medical College and Hospital, Taipei, Taiwan; ^2^ Department of Pathology, National Taiwan University, Medical College and Hospital, Taipei, Taiwan; ^3^ Tai-Cheng Stem Cell Therapy Center, National Taiwan University, Taipei, Taiwan

**Keywords:** soluble PD-L1, bone marrow plasma, multiple myeloma, autologous transplantation, prognosis

## Abstract

Autologous hematopoietic stem cell transplantation (AuHSCT) is standard in treating eligible multiple myeloma (MM) patients. However, the outcome after treatment is highly variable. We used ELISA to analyze the levels of soluble PD-L1 (suPD-L1) in bone marrow (BM) plasma from 61 patients with MM at 100 days after AuHSCT. Patients were classified into high (H) and normal-to-low (NL) groups depending on their suPD-L1 levels. Among patients who had a very good partial response (VGPR) or better after AuHSCT, those in the H-group had a shorter response period (RpSCT) as well as shorter overall survival (OS) than those in the NL-group. Multivariate analyses confirmed that a high suPD-L1 level and high-risk cytogenetic abnormalities are independent factors for RpSCT. Our data suggest that suPD-L1 in the BM plasma of MM patients who have VGPR or better after AuHSCT could be used as a biomarker to predict outcome.

## INTRODUCTION

High-dose melphalan followed by autologous hematopoietic stem cell transplantation (HDM/AuHSCT) was the standard therapy for transplant-eligible multiple myeloma (MM) patients in the conventional chemotherapy era [1, 2], and it is likely to remain as such in the novel agents era [3]. Nonetheless, nearly all MM patients eventually progress after HDM/AuHSCT with a widely variable progression-free survival (PFS) [2]. Several factors are associated with short survival after HDM/AuHSCT, such as a high proportion of S-phase cells, advanced disease stage according to the International Staging System (ISS) III, high levels of lactate dehydrogenase (LDH), high-risk cytogenetic abnormalities (CAs) [e.g., t(4;14) and del 17p], and lack of complete response (CR) after HDM/AuHSCT [4–8], among others. All such factors are related to myeloma cells (MCs), but not to the surrounding microenvironment, which is well known to contribute to survival and the development of drug resistance in MCs [9].

Recently, immunomicroenvironments have been shown to play a crucial role in several cancers [10]. Both immunoscores and immunosignatures in tumor microenvironments—mainly containing CD8^+^ cytotoxic T lymphocytes (CTLs), programmed cell death 1 (PD-1) and its ligand 1 (PD-L1), and interferon (INF)-γ, in addition to other cytokines and antibodies—can predict treatment response and outcome in various cancer types [10, 11]. A member of the CD28 receptor family, PD-1, as well as its ligands PD-L1 and PD-L2, play a fundamental role in maintaining T-cell homeostasis by restricting T-cell activation and proliferation [12]. The interaction of PD-1^+^ T-cells with PD-L1-expressing cells inhibits T-cell responses [10, 12]. Indeed, the expression of PD-L1 in tumor cells promotes T-cell tolerance, suppressing the secretion of stimulatory cytokines by T-cells, and inhibiting tumor-reactive CTLs [13, 14]. Interestingly, primary MCs express higher levels of PD-L1 [15–22]. In addition, T-cells and nature killer (NK) cells from MM patients also exhibit increased PD-1 expression [12, 17, 18, 22–24]. These immune effector cells are usually surrounded by PD-L1^+^ MCs; therefore, MCs may escape antitumor immunity [20]. Use of a checkpoint blockade, like an anti-PD-L1 monoclonal antibody, enhances immune effector cell-mediated anti-MM response [18, 20, 23]. Notably, following HDM/AuHSCT, the expression of PD-1 in T-cells decreases or returns to normal levels, suggesting that HDM/AuHSCT can reset the immunomicroenvironment of bone marrow (BM) [17]. Furthermore, synergistic effects have been observed from combining HSCT with PD-L1 blockade or other immune checkpoints inhibitors in a 5T33 murine MM model [16, 25].

Previous studies suggests that suPD-L1 might contribute to hematological malignancies and that suPD-L1 levels might correlate with treatment response and outcome in patients with diffuse large B-cell lymphoma and newly diagnosed MM [26, 27]. Here, we analyzed the levels of suPD-L1 in BM plasma samples from 61 patients who had received uniform anti-MM treatment, to investigate whether suPD-L1 levels after HDM/AuHSCT can be used as a progression biomarker.

## RESULTS

### Patients and response after HDM/AuHSCT

The salient clinical characteristics of the 61 patients at diagnosis are shown in Table 1. These 61 patients had received median four cycles (range: 2–8 cycles) and one cycle (range: 1–3 cycles) for the anti-MM induction treatment and mobilization of PBSCs, respectively. The median time from diagnosis of MM to day 0 of HDM/AuHSCT was eight months (range: 3–17 months). The response evaluation performed at 100 days after HDM/AuHSCT revealed that 14 (23%), 15 (25%), 20 (33%), and 12 patients (19%) reached sCR, CR, VGPR, and PR, respectively (Supplementary Table S1).

**Table 1:** Salient clinical characteristics of the 61 MM patients at diagnosis and the comparison between those who had high (H) and normal-to-low (NL) suPD-L1 levels in the BM plasma at 100 days after receiving HDM/AuHSCT

		suPD-L1		
Patients	All	H	NL	
N	61	18	43	*P*-value
Sex (M/F)	33/28	5/13	28/15	0.011
Age (yrs)*	53.6±8.4	52.7±9.5	53.9±8.0	0.598
DSS [N (%)]				0.097
I/II	30 (49)	12 (67)	18 (42)	
IIIa/b	31 (51)	6 (33)	25 (58)	
ISS [N (%)]				0.381
I/II	42 (69)	14 (78)	28 (65)	
III	19 (31)	4 (22)	15 (35)	
Isotype [N (%)]				0.273^#^
IgG	38 (62)	9 (50)	29 (67)	
IgA	9 (15)	2 (11)	7 (16)	
IgD	5 (8)	3 (17)	2 (5)	
Light-chain	9 (15)	4 (22)	5 (12)	
Kappa:Lambda ratio	2.2:1	3.5:1	1.9:1	0.381
Hemoglobin (gm/dL)*	9.5±2.7	9.3±2.5	9.6±2.8	0.728
White blood cell (x10^9^/L)*	6.7±4.4	6.9±5.5	6.6±3.9	0.809
Platelet (x10^11^/L)*	2.1±0.9	2.1±0.9	2.0±0.9	0.882
Creatinine (mg/dL)*	1.8±2.2	1.9±3.1	1.7±1.7	0.727
Calcium (μmol/L)*	2.3±0.4	2.2±0.2	2.3±0.4	0.441
LDH (IU/L)*	329±319	425±496	289±202	0.290
ALP (IU/L)*	183±199	169±144	189±217	0.742
CRP (mg/dL)*	1.1±1.8	0.8±1.1	1.2±2.1	0.509
Albumin (gm/dL)*	3.6±0.8	3.9±0.6	3.5±0.9	0.110
β_2_M (mg/L)*	5.8±5.6	6.2±7.5	5.6±4.7	0.747
Plasma cell in BM (%)*	57.9±30.6	63.6±32.8	55.8±29.9	0.386
High risk CAs [N (%)]	15 (25)	6 (33)	9 (21)	0.340^#^
CAs detected by CG	8 (13)	3 (17)	5 (12)	0.669^#^
FISH_t(4;14)	5 (8)	1 (6)	4 (9)	0.517^#^
FISH_t(14;16)	2 (3)	1 (6)	1 (2)	1.000^#^
FISH_del 17p	8 (13)	4 (22)	4 (9)	0.795^#^
EMD [N (%)]	13 (21)	5 (28)	8 (19)	0.499^#^

* mean±SD;

# Fisher's exact test

Abbreviations: ALP, alkaline phosphatase; BM, bone marrow; CAs, cytogenetic abnormalities; CG, conventional G-banding; CRP, C-reactive protein; DSS, Durie-Salmon staging; EMD, extramedullary disease; F, female; HDM/AuHSCT, high dose melphalan followed by autologous hematopoietic stem cell transplantation; ISS, International staging system; LDH, lactate dehydrogenase; M, male; MM, multiple myeloma; suPD-L1, soluble PD-L1;β_2_M, beta_2_-microglobulin

### Levels of suPD-L1

**Figure 1:**
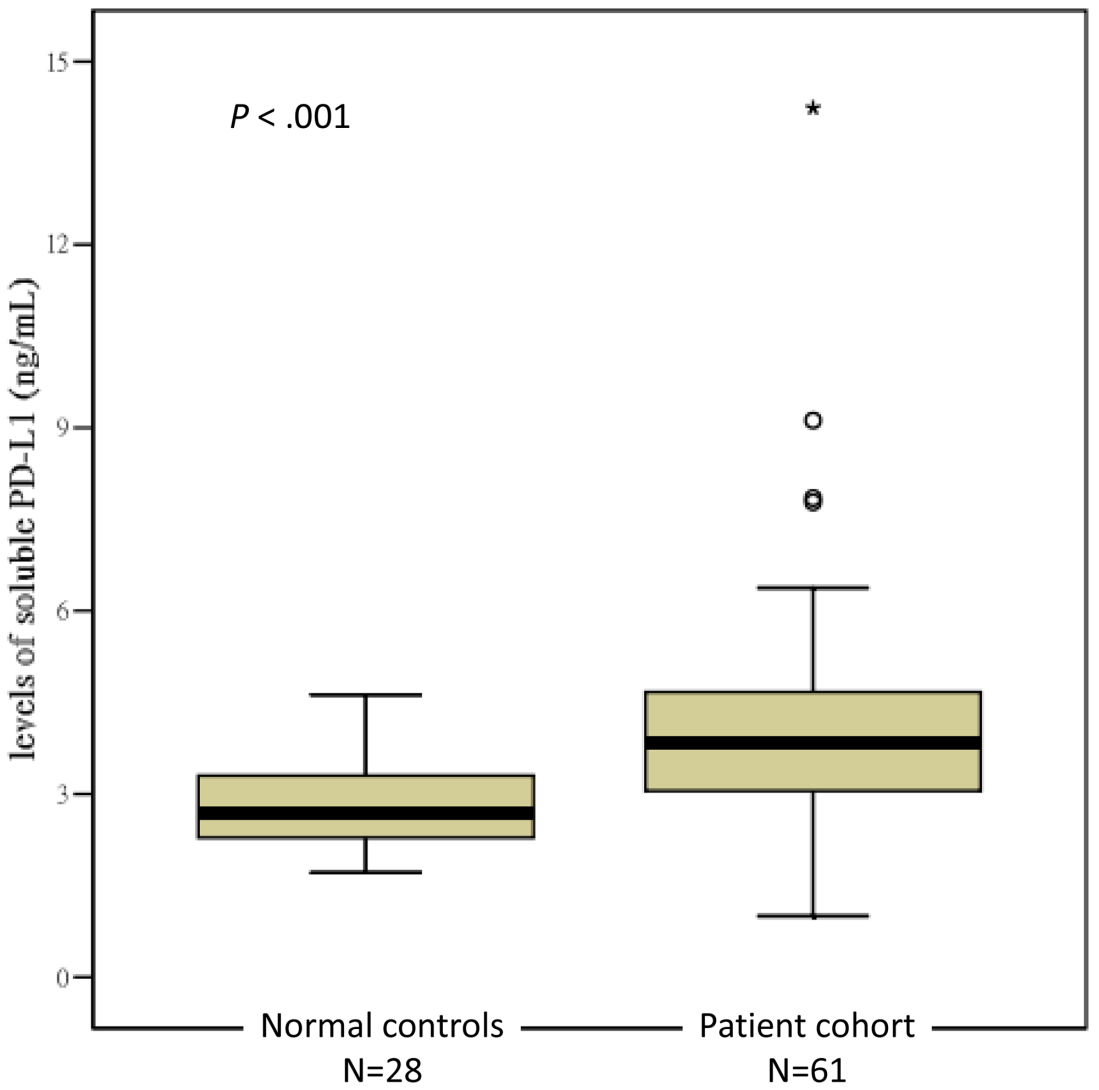
Levels of suPD-L1 between the normal controls and the experimental patients

**Figure 2:**
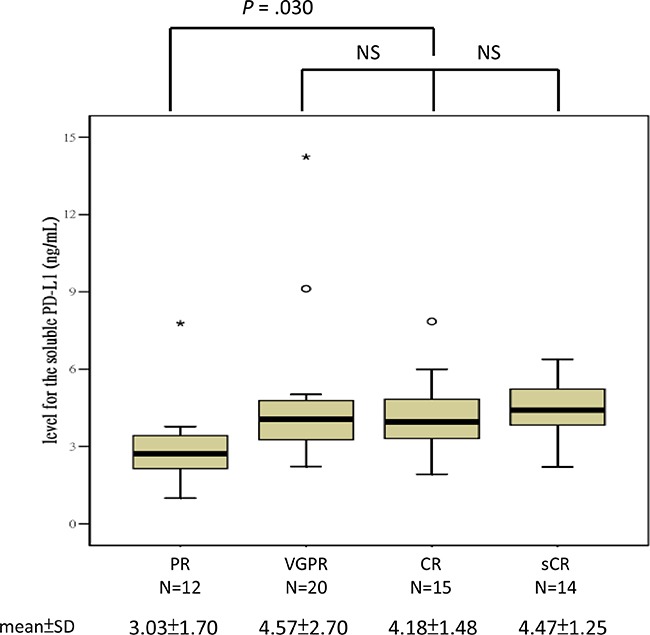
Levels of suPD-L1 among the various treatment response groups after HDM/AuHSCT

All plasma samples were analyzed at least in triplicate, and the mean intra-assay coefficient of variability was 4.2% and 7.6% in the normal controls and MM patients, respectively. The mean ± SD level of suPD-L1 in the 28 normal BM donors was 2.81 ± 0.77 ng/mL (range: 1.71–4.53 ng/mL). For the 61 MM patients, the mean ± SD level of suPD-L1 was 4.15 ± 2.01 ng/mL (range: 1.0–14.24 ng/mL). A comparison between the suPD-L1 levels of the normal controls and MM patients is presented in Figure 1; the difference was statistically significant (*P* < .001). In the normal controls, we observed no significant correlation between age and the level of suPD-L1 (Pearson's correlation: 0.158, *P* = .421; Spearman's correlation: 0.168, *P* = .394). The levels of suPD-L1 for the patients who had PR were significantly lower than those for patients who had VGPR or better (mean ± SD: 3.03 ± 1.70 ng/mL vs 4.42 ± 1.99 ng/mL, respectively; *P* = .030) (Figure 2). By applying the cutoff of 4.54 ng/mL, selected from the uppermost level of the normal controls, we determined that 18 (30%) patients had suPD-L1 levels higher than 4.54 ng/mL; these patients constituted the high-suPD-L1 group (H group). The remaining 43 (70%) patients constituted the normal-to-low group (NL group) (≤4.54 ng/mL). The comparison between the clinical characteristics at diagnosis of the H and NL groups is shown in Table 1; no differences were found between these groups, except that the H group had a higher proportion of female patients compared with the NL group (*P* = 0.011). The response distribution after HDM/AuHSCT between the H and NL groups is shown in Supplementary Table S1; the difference was not statistically significant.

### Association of high suPD-L1 level and shorter response period in patients with VGPR or better after HDM/AuHSCT

**Figure 3:**
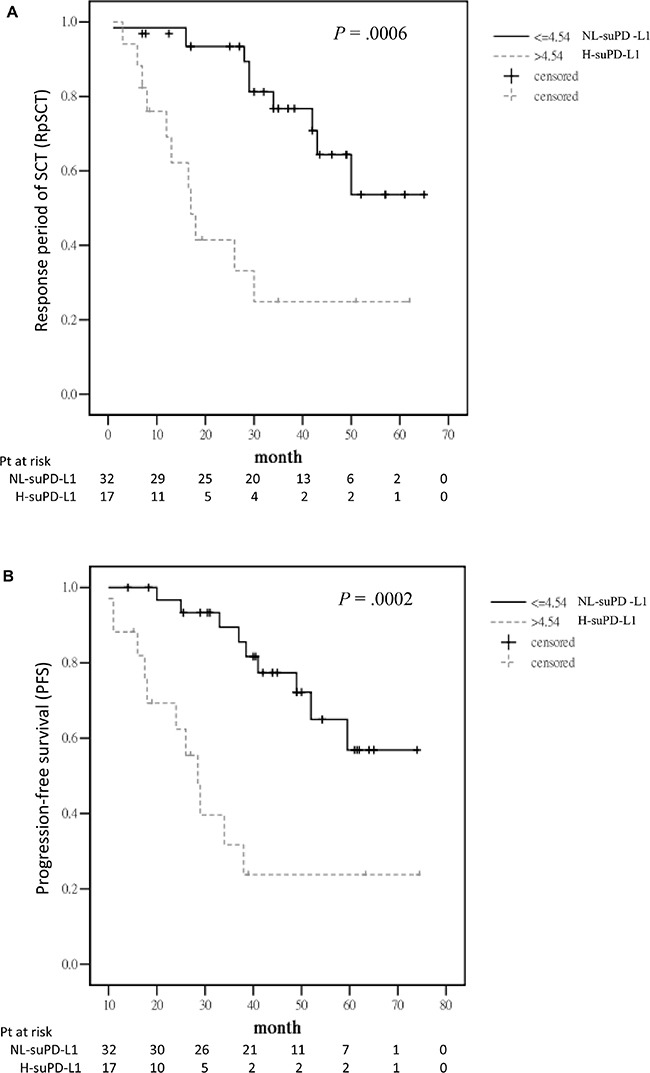

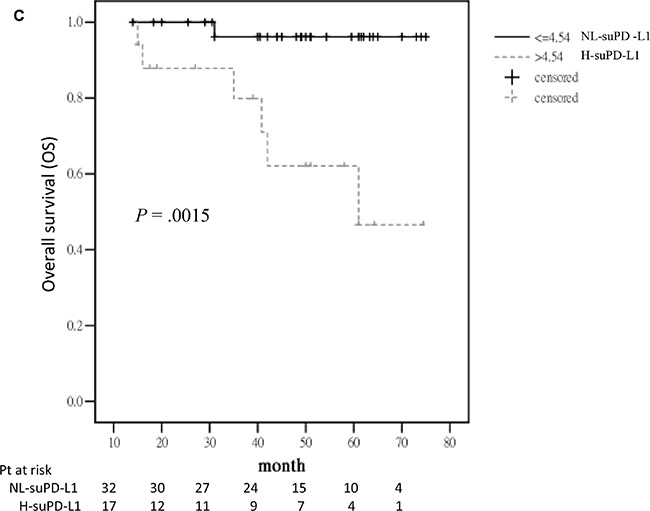
Outcome for the 49 patients with VGPR or better after HDM/AuHSCT **A.** RpSCT between the H and NL groups measured by suPD-L1 levels. **B.** PFS for front-line anti-MM treatment between the H and NL groups. **C.** OS between the H and NL groups.

The response period for HDM/AuHSCT (RpSCT), defined from day 0 of HDM/AuHSCT to the date of documented progressive disease (PD), for patients who only had PR after single HDM/AuHSCT was shorter than that for those who had VGPR or better [median: 11 months (95% CI: 0–26.28) vs 50 months (95% CI: 35.8–64.2); *P* = .0001]. Among the 49 patients who had reached VGPR or better, patients in the H group had a shorter RpSCT than those in the NL group [median: 17 months (95% CI: 14.3–19.8) vs not reached (NR); *P* = .0006] (Figure 3A). The PFS of the front-line anti-MM treatment was also shorter for patients in the H group than for those in the NL group [median: 28.5 months (95% CI: 23.6–33.4 months) vs NR; *P* = .0002] (Figure 3B). After a median follow-up period of 50 months (95% CI: 47.7–52.4 months), the median OS was found to be shorter in the H group than in the NL group (61 months vs NR; *P* = .0015) (Figure 3C).

### Multivariate analysis confirmation of high suPD-L1 as a negative independent predictor for the RpSCT and OS

Among the 49 patients who had VGPR or better after HDM/AuHSCT, several factors associated with the RpSCT and OS detected by Cox regression univariate analysis are presented in Tables 2 and 3, respectively. After a multivariate analysis, the high suPD-L1 (>4.54 ng/mL) was confirmed to be a negative independent factor for RpSCT (Hazard ratio: 4.322; 95% CI: 1.708–10.936; *P* = .002) and OS (Hazard ratio: 9.181; 95% CI: 1.069–78.820; *P* = .043). The other significant factor, high-risk CAs, remained independent for both the RpSCT and OS (Tables 2 and 3). The results of comprehensive uni- and multivariate Cox regression analyses for PFS and OS are presented in Supplementary Tables S2 and S3.

**Table 2:** Cox regression analysis among levels of suPD-L1 and salient clinical features at diagnosis associated with progression of HDM/AuHSCT in the 49 MM patients with VGPR or better after HDM/AuHSCT

	Univariate analysis	Multivariate analysis
Item	Hazard Ratio (95% CI)	
suPD-L1 (ng/mL)		
<= 4.54	ref	ref
> 4.54	4.295 (1.746-10.565)**	4.322 (1.708-10.936)**
Hb >= 10 gm/dL	0.352 (0.127-0.981)*	-
High risk CAs	4.243 (1.605-11.218)**	4.268 (1.547-11.771)**

* Statistical significance, p<0.05;

** p<0.01

Abbreviations: CAs, cytogenetic abnormalities; CI, confidence interval; Hb, hemoglobin; HDM/AuHSCT, high dose melphalan followed by autologous hematopoietic stem cell transplantation; suPD-L1, soluble PD-L1; VGPR, very good partial response

**Table 3:** Cox regression analysis among levels of suPD-L1 and salient clinical features at diagnosis associated with overall survival in the 49 MM patients with VGPR or better after HDM/AuHSCT

	Univariate analysis	Multivariate analysis
Item	Hazard Ratio (95% CI)	
suPD-L1 (ng/mL)		
<= 4.54	ref	ref
> 4.54	13.796 (1.658-114.818)*	9.181 (1.069-78.820)*
LDH > ULN	7.453 (1.636-33.959)**	-
PC > 50%	9.477 (1.110-80.955)*	-
High risk CAs	18.759 (2.183-161.225)**	10.406 (1.186-91.330)*

* Statistical significance, p<0.05;

** p<0.01

Abbreviations: CAs, cytogenetic abnormalities; CI, confidence interval; HDM/AuHSCT, high dose melphalan followed by autologous hematopoietic stem cell transplantation; LDH, lactate dehydrogenase; MM, multiple myeloma; PC, plasma cells; ref, reference; suPD-L1, soluble PD-L1; ULN, upper limit of normal range

### Optimal cutoff for suPD-L1 derived from the ROC curve

For the RpSCT, the optimal cutoff for suPD-L1 derived from the ROC curve was 4.55 ng/mL (with an AUC of 0.5672), and this provided positive and negative predictive values of 55% and 79%, respectively (Supplementary Figure S1). For OS, the optimal cutoff for suPD-L1 was 4.52 ng/mL (with an AUC of 0.8061), and this provided positive and negative predictive values of 86% and 71%, respectively (Supplementary Figure S2).

### Correlation between suPD-L1 and the other immunological parameters

Among the 61 MM patients, we evaluated several immunological parameters such as incidence of total recovery of immunoparesis and absolute counts of neutrophils, lymphocytes, and monocytes in the PB taken at the time of BM examination; however, we determined no correlations among these factors and the level of suPD-L1 (Supplementary Table S4). Among the 49 patients who had VGPR or better after HDM/AuHSCT, we observed no differences concerning these immunological parameters between the H and NL groups (Supplementary Table S5).

### Cell density of CD8^+^ CTLs

Among the 61 patients, 56 BM biopsied samples were adequate for measurement of the cell density of CD8^+^ CTLs, which was 7.22 ± 5.69% (mean ± SD) with a range from 1.50% to 38.33%. The findings between independent observers showed good correlation (Pearson's correlation, 0.81; *P* < .001). The representative CD8^+^ CTLs within BM identified by the IHC staining were shown in Supplementary Figure S3A and there were no differences among the mean cell density of CD8^+^ CTLs between patients with various responses (PR vs VGPR vs CR vs sCR, 7.60% vs 7.22% vs 5.31% vs 9.22%, respectively; *P* = .387). Among the different cell density scores of CD8^+^ CTLs, there were no differences on RpSCT (Supplementary Figure S3B) or other immunological parameters, including the levels of suPD-L1 (Supplementary Table S6).

## DISCUSSION

Our study demonstrates for the first time that the levels of suPD-L1 in BM plasma at 100 days after HDM/AuHSCT in MM patients with VGPR or better are significantly and independently associated with patient outcome. By applying a cutoff (>4.54 ng/mL) determined by the upper limit of our normal controls, we observed that approximately 30% of our patients had higher suPD-L1 levels (i.e., the H group). These patients had a shorter PFS and OS compared with those who had lower suPD-L1 levels (i.e., NL group). Notably, the optimal cutoff generated from our ROC curve analysis was nearly the same level as that determined by the normal controls, which showed moderate AUC, providing fair-to-good model fitness as well as positive and negative predictive values for both PFS and OS (Supplementary Figures S1 and S2). SuPD-L1 might be able to inhibit the function of CTLs by binding to PD-1 on T-cells, similar to its membrane counterpart (mPD-L1) [10, 33], thereby preventing host immunity from eradicating the minimal residual disease (MRD) or preventing immediate resurgence of MCs. Supporting this notion, a previous study *in vitro* indicated that suPD-L1 could suppress the proliferation of T-cells and induce a Th2 immune response [34], suggesting that suPD-L1 can act as a fully functional molecule. This study also supports the hypothesis that suPD-L1 could be used as a biomarker to predict the outcome of MM patients after HDM/AuHSCT. In a recent study, a subpopulation of T-cells with an exhaustion/senescence phenotype identified at 3 months after HDM/AuHSCT was determined to be an early distinguishing feature ahead of clinical relapse [35]. Taken together, these data indicate that the immunomicroenvironment in BM after HDM/AuHSCT in MM patients, particularly at a low tumor mass burden, plays a role in determining the outcome. Our multivariate analyses confirmed that high suPD-L1 was an independent prognostic factor. Another independent factor was high-risk CAs, which is a well-known prognostic factor in MM patients [4]. Furthermore, suPD-L1 remained an independent factor, even in the presence of other prognostic factors including age over 60 years, high level of LDH and ISS III (data not shown). However, the female predominance in the H group is inexplicable.

Our observations also raise the question as to how suPD-L1s are produced. Previous studies have shown that suPD-Ll might be produced when matrix metalloproteinases (MMPs) cleave the extracellular fraction of mPD-L1 [33, 36]. A similar process was observed for soluble B7-H3, another co-stimulatory molecule on antigen presenting cells (APCs) that could be released from cell membranes through MMPs cleavage [37]. In support of this notion, suPD-L1 was detectable in supernatants from mPD-L1^+^ cells but not in those from mPD-L1^−^ cell lines [33]. However, whether such cleavage occurs randomly or is regulated by specific mechanisms remains to be determined. Moreover, whether suPD-L1 can be produced by other mechanisms, such as alternative splicing, is unclear [38, 39].

No association has been observed between suPD-L1 levels and tumor PD-L1 expression in patients with DLBCL and renal cell carcinoma [26, 40]; therefore, nonmalignant cells in the tumor microenvironment may produce suPD-L1 as well. Whether non-MCs in BM produce suPD-L1 remains unknown, but there may be several candidates that can express mPD-L1 and produce suPD-L1 through MMPs cleavage, including myeloid-derived suppressor cells (MDSCs), tissue histiocytes, toll-like receptor APCs, and plasmacytoid DC [41–44]. Among these, MDSCs in the BM of MM patients remain as PD-L1^+^ even at remission because of persistent hypoxia in BM [18, 20, 45]. Furthermore, the frequency of MDSCs in the BM of MM patients appears to be higher at remission than at diagnosis or relapse [20]. Notably, ibrutinib, a bruton tyrosin kinase inhibitor, was found to deplete MDSCs in tumor models and to synergize with anti-PD-L1 therapy [46]. Accordingly, measuring suPD-L1 in BM plasma, as conducted in this study, may be more useful than measuring mPD-L1 on MCs alone. Moreover, the plasma obtained from BM is likely to be more relevant than that from PB because it is closer to the immunomicroenvironment surrounding MCs [19, 20].

Similar to the findings of other studies [6, 8], approximately 20% of our MM patients had PR only after HDM/AuHSCT, and their outcome was poor compared with that of patients who had VGPR or better. However, the reason suPD-L1 was significantly lower in patients with PR after HDM/AuHSCT than in those who had VGPR or better is unclear (Figure 2). Considering that syndecan-1/CD138 shedding induced by apoptosis of MCs after chemotherapy [47], we hypothesize that killing fewer MCs would induce less mPD-L1 shedding, thereby resulting in lower levels of suPD-L1 within the matrix.

The various mechanisms that might contribute to relapse/progression of MM after HDM/AuSCT can be divided into 1) those related to tumor biology, e.g. tumor resistance and aggressiveness, and 2) those related to the status of the BM microenvironment, e.g. immune competence within the BM microenvironment. In some of our patients who had PR already before HDM/AuSCT, the response could not be further intensified after HDM/AuSCT, suggesting treatment resistance and high-risk tumor biology. On the other hand, in patients with VGPR either before or after the HDM/AuSCT, the tumor immunomicroenvironment may play a more predominant role than tumor biology in determining relapse/progression.

Our study has several limitations. First, this study was a retrospective analysis with a heterogeneous background and a limited sample size. However, the patients were treated in a single institute under a homogenous protocol, which might have minimized the variation in treatment effects among individual patients. Second, this study lacked a sensitive MRD detection approach for comparison either by next-generation flow cytometry or sequencing-based techniques, which are powerful prognostic factors [48]. Nevertheless, we employed four-color flow cytometry for any aberrant immunophenotypic MCs according to the updated recommendation by IMWG [29]. Third, no sex- or age-matched controls were employed in this study. Notably, similar to another study [27], the distribution of suPD-L1 in our normal controls did not correlate with age. However, a correlation between suPD-L1 and age in healthy donors was reported in a different study [49]. Forth, our study lacked a longitudinal follow-up for suPD-L1 at various clinical status levels. Finally, statuses were not identified for other checkpoints such as TIM-3, LAG-3, and CTLA-4, all of which might play a synergistic role with PD-L1 in immune inhibition [10, 25]. Although analyzing more samples with longer follow-up times could further substantiate our findings, our data indicate that suPD-L1 can serve as a biomarker to predict the outcome of MM patients with VGPR or better after HDM/AuHSCT.

## MATERIALS AND METHODS

### Patients and BM plasma

During July 2009–January 2015, a total of 61 patients with MM who had responded, terms of partial response (PR) or better, to uniform anti-MM induction treatment at our institute followed by single autologous transplantation were enrolled. EDTA anticoagulated BM plasma samples (10–20 mL) obtained upon routine BM evaluation, including BM needle aspiration and biopsy, performed at 100 days after the transplantation, were collected and processed as described previously [28]. In addition, BM plasma samples collected from 28 healthy BM donors (comprising 13 men and 15 women with a median age of 48 years: range, 13–67 years), during 2014–2015 were used as normal controls. Among these aspirated BM blood samples, the first drawn sample, if available, was the preferred one to use. This study and its consent procedure were approved by the National Taiwan University Hospital Research Ethics Committee (NTUHREC: 201505162RINC). Written informed consent was obtained from all study participants in accordance with the Declaration of Helsinki.

### Induction regimen, treatment response and outcome

The uniform anti-MM induction treatment for transplant-eligible patients at our institute was as previously described [28]. Namely BTD+Cy: bortezomib (B) [1.3 mg/m^2^ (sc or iv) at days 1, 4, 8, and 11] plus thalidomide (T) (100–200 mg/d) and oral dexamethasone (D) (20–40 mg/d at days 1–4) and oral cyclophosphamide (Cy) (100 mg at days 1–4) in a 21-day cycle. Upon reaching PR or better after induction, autologous PB stem cells (PBSCs) were mobilized with high-dose Cy (2 gm/m^2^) plus G-CSF (5–10 μg/Kg) and collected. HDM (140–200 mg/m^2^) was employed to condition AuHSCT. T 50–100 mg/d was provided for post-HDM/AuHSCT maintenance. The treatment outcomes, comprising CR, stringent CR (sCR), PR, very good partial response (VGPR), relapse, progressive disease (PD), PFS, and overall survival (OS) measured from diagnosis, were reevaluated in each patient on the basis of the IMWG consensus criteria [29]. The response period for HDM/AuHSCT (RpSCT) was defined from day 0 of HDM/AuHSCT to the date of documented PD.

### Enzyme-linked immunosorbent assay (ELISA) for suPD-L1

The suPD-L1 levels in the BM plasma samples were measured using an enzyme-linked immunosorbent assay (ELISA) (PDCD1LG1 ELISA kit; USCN Life Science, Wuhan, China), according to manufacturer instructions. In brief, 100 μL plasma sample or standard protein was added to each well and incubated at 37°C for 2 h. Detection reagent A (100 μL) was added and incubated at 37°C for 1 h. Next, the wells were incubated with detection reagent B at 37°C for 30 min, and subsequently with 90 μL substrate solution at room temperature for 20 min and protected from light. The wells were finally incubated with 50 μL stopping solution. The wells were then placed on an ELISA plate reader (Perkin Elmer, CA, USA), and the absorbance of each well was recorded at 450 nm. Standard PD-L1 protein with serial dilutions (0–10 ng/μL) was used as a standard in each experiment. Each sample and standard protein was analyzed in triplicate; the minimum detection level of suPD-L1 was 0.156 ng/mL. The suPD-L1 levels (ng/mL) were calculated by linear regression.

### Immunohistochemical stain for CD8^+^ CTLs

The procedures for immunohistochemical (IHC) staining in our laboratory were executed as described in a previous study [30], but optimized for the current study. Briefly, BM biopsied samples were fixed in 10% neutral buffered formaldehyde for at least 24 h, decalcified with Shandon TBO-2 decalcifier (Thermo Scientific, US) for 2 h, and embedded in paraffin. Paraffin-embedded BM tissue sections measuring 4–5 μm were deparaffinized in xylene, rehydrated with ethanol, and rinsed in PBS. After deparaffinization and rehydration, the slides were placed in the target retrieval solution (S1700, Dako, Denmark) and heated (90–99°C) for 40 min. Endogenous peroxidase was then blocked with 3% hydrogen peroxide for 10 min (Dako, Denmark); nonspecific protein binding was blocked with 3% BSA and 10% FBS (Corning NY, USA) and was then incubated at room temperature for 60 min. After blocking, the slides were incubated overnight with the primary antibody at 4°C. The primary antibody was used to identify the CD8 (1:100) (clone IF6; Novocastra, Newcastle upon Tyne, UK). For the maximal cell density of the CD8^+^ CTLs, we first identified three locations with the maximum number of CD8^+^ CTLs under a low-power field (100x), then switched to a high-power field (HPF, 600x), counting the CD8^+^ CTLs in each field (number of CD8^+^ CTLs/total amount of nucleated cells). The cell density of the CD8^+^ CTLs was averaged from the three counted areas. Scoring cutoffs for the averaged maximal density of CD8^+^ CTLs was set as follows: score 0, 0% to <= 10% positive cells; score 1, > 10% to <= 20% positive cells; score 2, > 20% positive cells.

### Flow cytometry

In our laboratory experiment, four-color flow cytometry was employed to detect the clonotypic plasma cells in the BM. The panels of four-color fluorescent monoclonal antibodies (Becton Dickinson, BD Bioscience, San Jose, CA) are listed as follows: CD45-PerCP/CD38-PE/CD138-APC/cytoplasmic Kappa-FITC; CD45-PerCP/CD38-PE/CD138-APC/cytoplasmic Lambda-FITC; and CD45-PerCP/CD117-PE/CD138-APC/CD20-FITC. Additionally, three-color panels were used, listed as follows: CD45-PerCP/CD38-PE/CD56-FITC; CD45-PerCP/CD19-PE/surface Kappa-FITC; and CD45-PerCP/CD19-PE/surface Lambda-FITC. The experimental procedures were performed on cells isolated from BM by lysing and washing, as described previously [31]. Briefly, for each analysis, at least 100,000 events with the appropriate ratio of forward scatter to side scatter were collected and analyzed on a FACS Canto II flow cytometer (BD Bioscience, San Jose, CA) by using WinList software. Optimally titrated antibodies were added to 100 μL BM aspirates and incubated for 20 min at room temperature in the dark. After incubation, red blood cells were lysed by ammonium chloride, and the remnant targeted cells were fixed to stabilize cell membranes and prevent dissociation of the fluorescent monoclonal antibodies. Subsequently, all cells were permeabilized using a commercial kit (Cytofix/CytopermTM Fixation/Permeabilization; BD Bioscience, San Jose, CA) for intracellular staining.

### Statistics

Chi squared or Fisher's exact tests were used for intergroup comparisons of the discrete variables. A two-sample *t* test was employed for intergroup comparisons of the means. Pearson's or Spearman's correlation tests were used to determine the correlation between continuous variables. Kaplan–Meier survival curves were constructed to estimate the PFS, OS, and RpSCT, and the intergroup differences were compared using a log-rank test. In the analyses, identified salient variables for the clinical and laboratory data were categorized as previously described [28] and are listed as follows: age ≥ 60 years; stage ≥ Durie–Salmon Stage III; stage ≥ International Staging System III; nonIgG isotype; Hb < 10 g/dL; WBC < 4.0 × 10^9^/L; PLA < 1.5 × 10^11^/L; LDH ≥ upper limit of normal range (ULN); ALP ≥ ULN; Ca ≥ 2.4 μmol/L; Cr ≥ 2.0 mg/dL; and C-reactive protein (CRP) ≥ ULN (0.8 mg/dL). High-risk CAs represented clonal changes detected by the conventional G-banding technique, and/or t (4;14), t (14;16), del (17p) detected by fluorescence *in situ* hybridization, performed as previously described [32]. Factors that provided statistically significant predictive power in the univariate analysis were further subjected to multivariate regression analysis of the linear, logistic, or Cox type with backward elimination and stepwise entering. Regarding the RpSCT and OS, the optimal cutoff value of suPD-L1 was selected with Youden's index by using a receiver-operating characteristic (ROC) curve; the area under the ROC curve (AUC) was then calculated. All directional *P* values were two-tailed, and *P* ≤ 0.05 was considered significant for all tests. All analyses were performed using SPSS Version 19.0 software (Chicago, IL, USA).

## SUPPLEMENTARY FIGURES AND TABLES


