# Supplementary material for: Soluble PD-L1: A biomarker to predict progression of autologous transplantation in patients with multiple myeloma
Source: Oncotarget. 2016 Aug 23;7(38):62490–502. doi: 10.18632/oncotarget.11519 (PMC5308741; doi:10.18632/oncotarget.11519)
Supplement: Supplementary file 1 [file oncotarget-07-62490-s001.pdf]

Soluble PD-L1: A biomarker to predict progression of autologous transplantation in patients with multiple myeloma

SUPPLEMENTARY FIGURES AND TABLES

Outcome = PFS

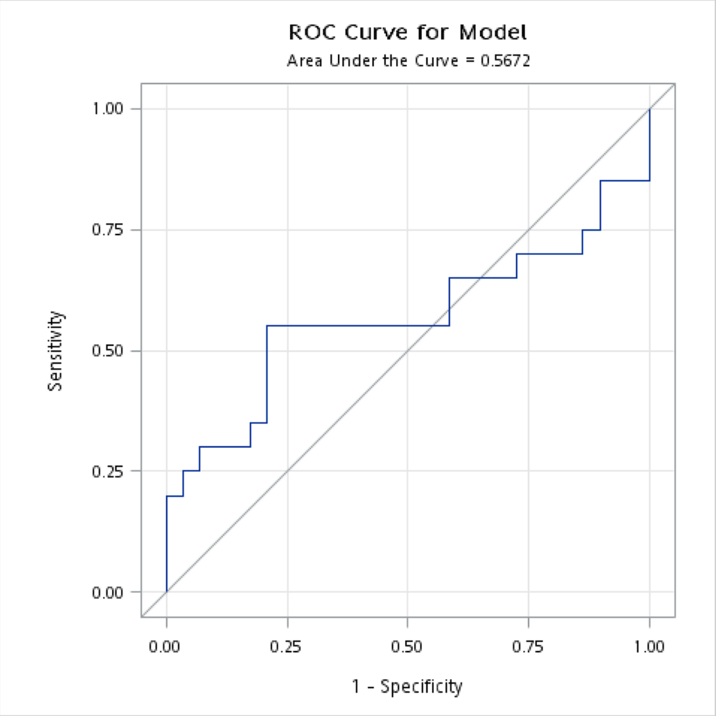

AUC = 0.5672

Best su_PDL1 cut-off = 4.55、Sensitivity = 0.55、Specificity = 0.7931

Table of PFS_event by prediction			
**PFS_event**	**Prediction***		
	**H-group**	**NL-group**	**Total**
**1**	11	9	20
**0**	6	23	29
**Total**	17	32	49
**Frequency Missing = 28**			

*. *H-group: su_PDL1*≥ 4.55；*NL-group: su_PDL*<4.55

Accuracy=(11+23)/49=69.39%

Sensitivity=11/17=64.71%

Specificity=23/32=71.88%

Positive Predictive Value (PPV) = 11/20=55%

Negative Predictive Value (NPV) = 30/37=79%

Supplementary Figure S1: ROC curve generated for PFS among the 49 patients with VGPR or better after HDM/AuHSCT.

Outcome = Overall Survival

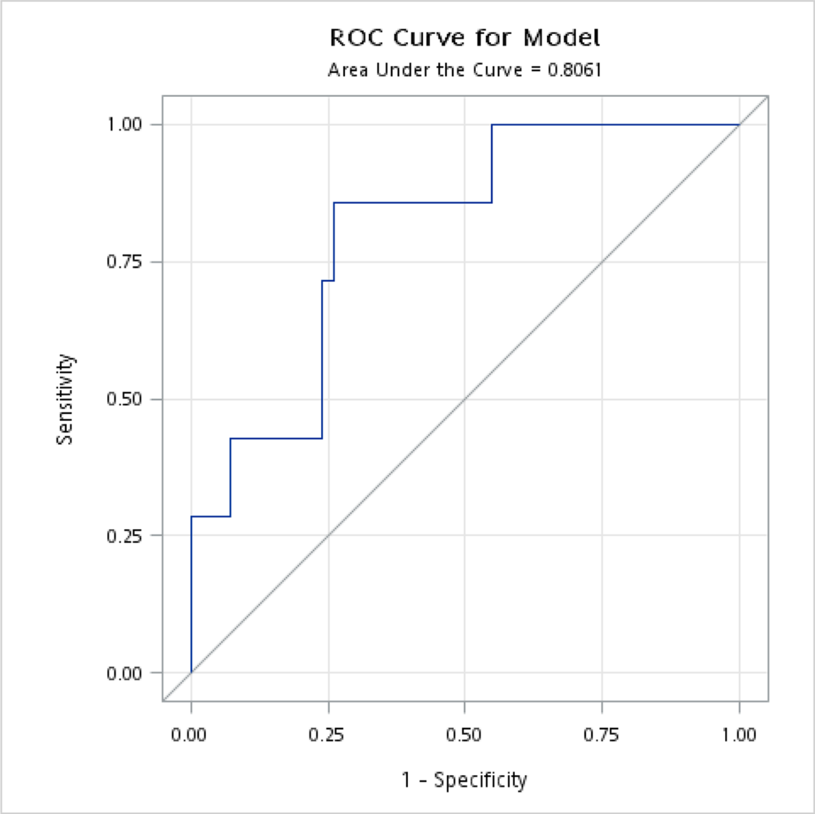

AUC = 0.8061

Best su_PDL1 cut-off = 4.52、Sensitivity = 0.8571、Specificity = 0.7381

Table of outcome by prediction			
**outcome**	**Prediction***		
	**H-group**	**NL-group**	**Total**
**0(dead)**	6	1	7
**1(survive)**	12	30	42
**Total**	18	31	49
**Frequency Missing = 28**			

**. H-group: su_PDL1*≥ 4.52 *; NL-group: su_PDL*<4.52

Accuracy=(6+30)/49=73.47%

Sensitivity=6/18=33.33%

Specificity=30/31=96.77%

Positive Predictive Value (PPV) = 12/42=86%

Negative Predictive Value (NPV) = 30/42=71%

Supplementary Figure S2: ROC curve generated for OS among the 49 patients with VGPR or better after HDM/AuHSCT.

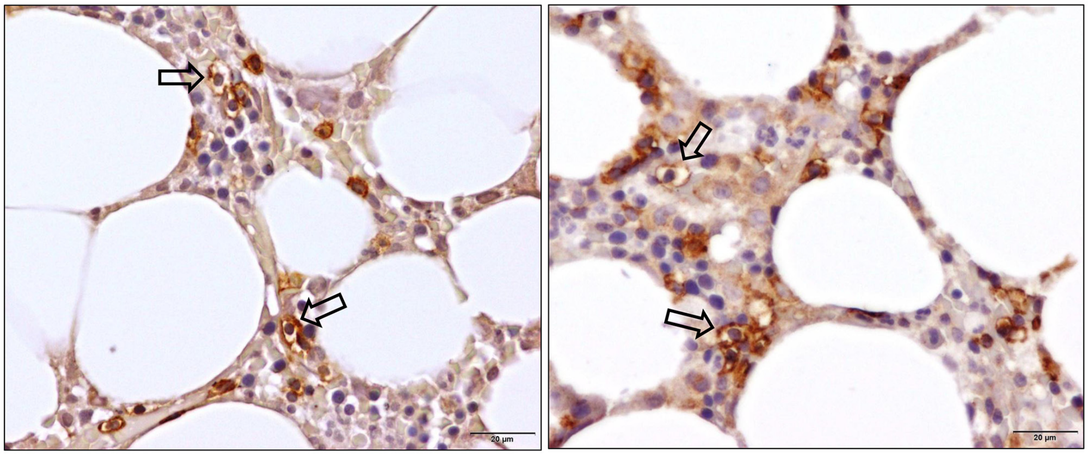

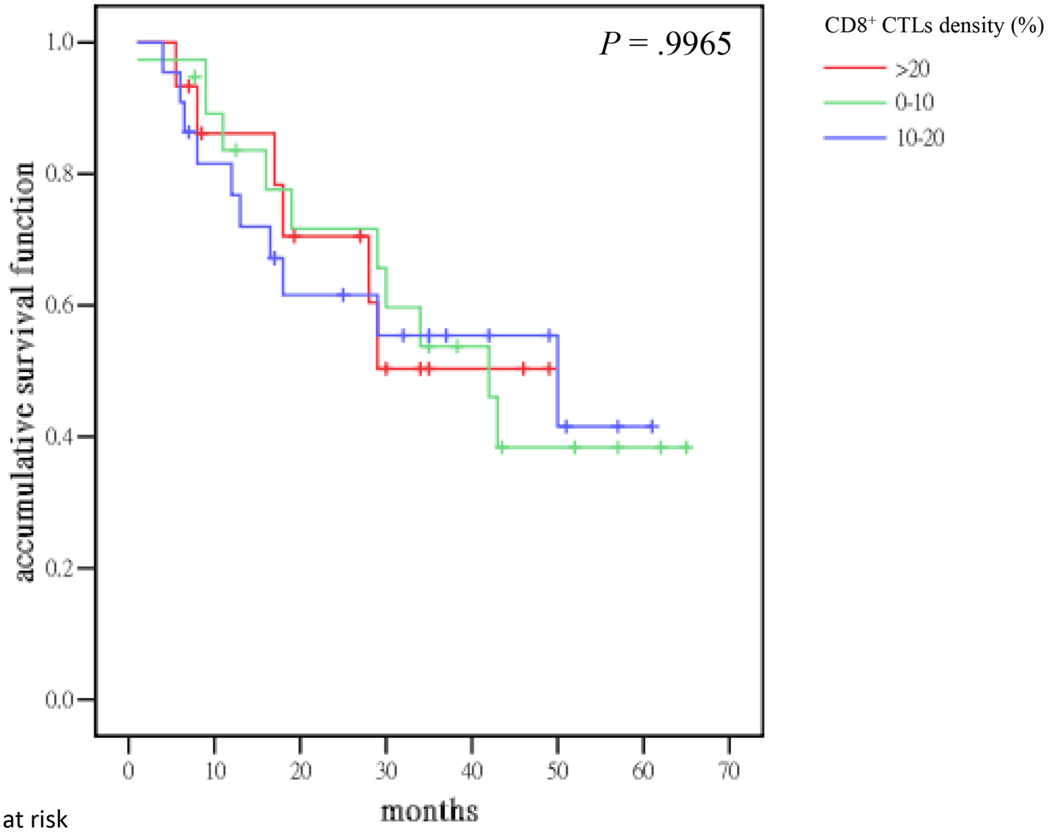

Pt at risk								
>20	15	10	6	4	1	0	0	0
0-10	19	16	12	10	6	3	1	0
10-20	22	16	10	8	5	3	1	0

Supplementary Figure S3: IHC staining for CD8+ CTLs. A. CD8+ CTLs in BM are identified by typical membrane brown staining (arrows) in two representative patients (400x; bar scale: 20 µm); **B.** RpSCT among the different cell density scores of CD8+ CTLs in BM.

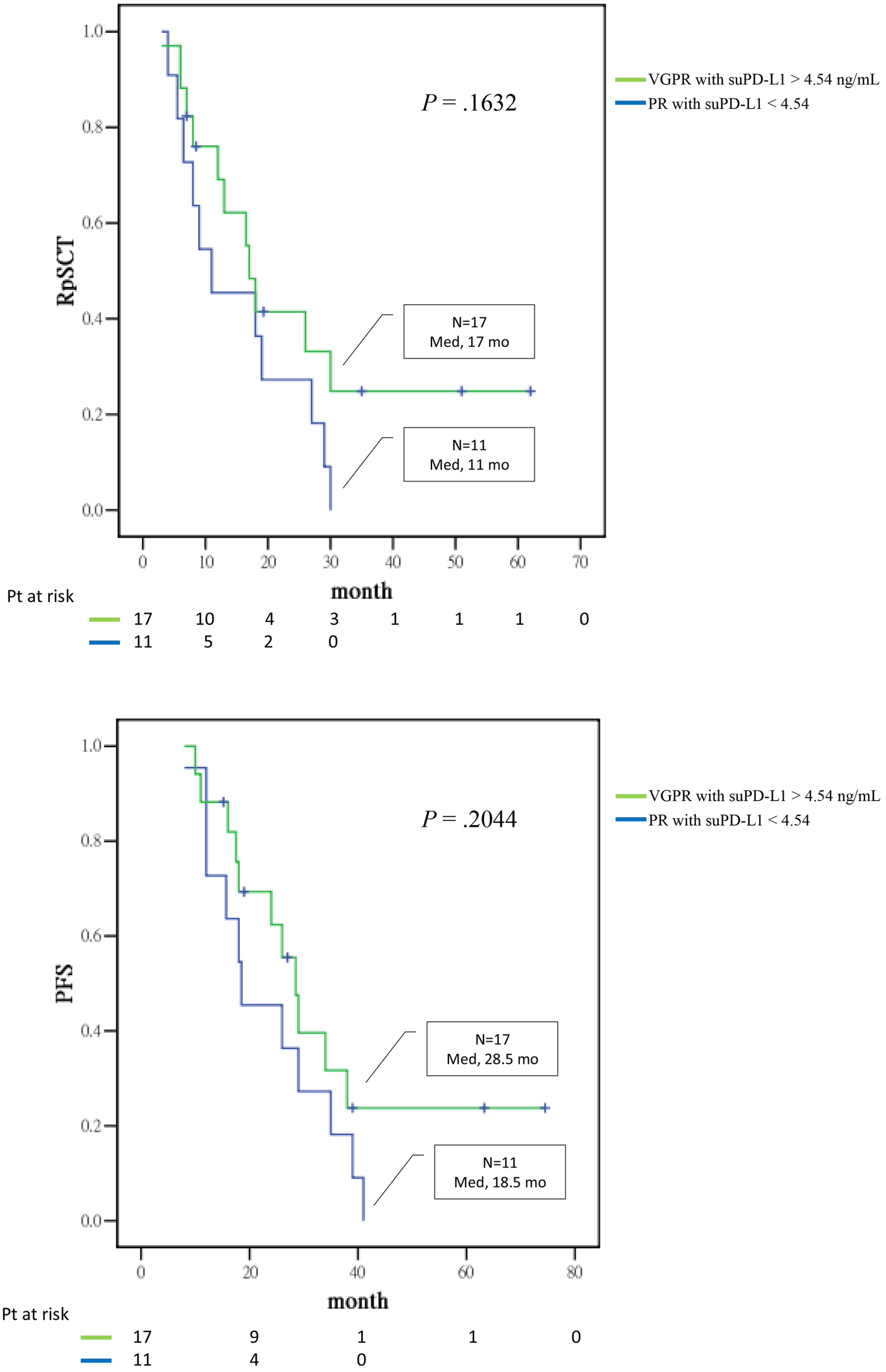

Supplementary Figure S4: Outcome between patients who had VGPR after HDM/AuSCT with suPD-L1 > 4.54 ng/mL and those who had PR after HDM/AuSCT with suPD-L1 < 4.54. A. response period of stem cell transplantation (RpSCT); **B.** progression-free survival (PFS) (*Continued*).

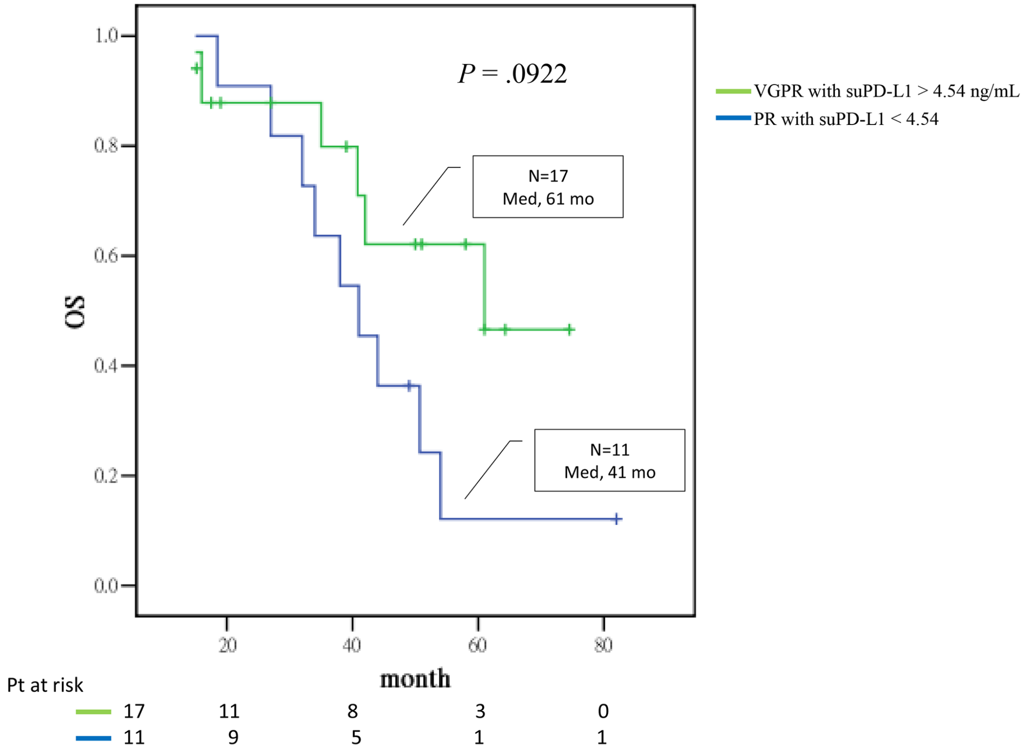

Supplementary Figure S4: (*Continued*) Outcome between patients who had VGPR after HDM/AuSCT with suPD-L1 > 4.54 ng/mL and those who had PR after HDM/AuSCT with suPD-L1 < 4.54. C. overall survival (OS).

Supplementary Table S1: Response to HDM/AuHSCT among high (H) and normal-to-low (NL) suPD-L1 groups

Patients	All	suPD-L1		
		H	NL	
N	61	18	43	*P*-value
Response after HDM/ AuSCT [N (%)]				0.257#
sCR	14 (23)	6 (33)	8 (19)	
CR	15 (25)	4 (22)	11 (26)	
VGPR	20 (33)	7 (39)	13 (29)	
PR	12 (19)	1 (6)	11 (26)	

#. Fisher's exact test

Abbreviations: CR, complete response; H, high; HDM/AuHSCT, high dose melphalan followed by autologous hematopoietic stem cell transplantation; NL, normal to low; PR, partial response; sCR, stringent CR; VGPR, very good PR

Supplementary Table S2: Cox regression analysis among levels of suPD-L1 and salient clinical features at diagnosis associated with progression of HDM/AuHSCT in the 49 patients with VGPR or better after HDM/AuHSCT

	Univariate analysis	Multivariate analysis
Item	Hazard Ratio (95% CI)(*P*-value)	
sPD-L1 level (ng/mL)		
<= 4.54	ref	ref
> 4.54	4.295 (1.746-10.565)**(0.002)	4.322 (1.708-10.936)**(0.002)
Sex		
Male	ref	-
Female	1.692 (0.700-4.090) (0.243)	-
Age >= 60 years	1.937(0.730-5.140)(0.184)	-
Hb >= 10gm/dL	0.352 (0.127-0.981)*(0.046)	-
WBC >= 4000/uL	0.697 (0.231-2.103)(0.522)	-
Pla >= 150k/uL	0.658 (0.252-1.718)(0.392)	-
LDH > ULN	1.988 (0.701-5.632)(0.196)	-
Alb >= 3.5 gm/dL	0.887 (0.338-2.328)(0.807)	-
Cr >= 2.0 mg/dL	0.711 (0.207-2.436)(0.587)	-
ALP > ULN	0.620 (0.233-1.647)(0.337)	-
Ca > 2.4	1.174 (0.355-3.886)(0.793)	-
CRP > 0.8 mg/dL	1.396 (0.535-3.645)(0.496)	-
ISS III vs ISS I/II	1.029 (0.393-2.690)(0.954)	-
DSS IIIa/b vs DSS I/II	1.675 (0.669-4.188)(0.270)	-
EMD	1.473 (0.525-4.136)(0.462)	-
With amyloidosis	0.043 (0.000-35.040)(0.357)	-
Light chain type	0.674 (0.244-1.860)(0.446)	-
IgG type	0.711 (0.295-1.714)(0.447)	-
PC > 50%	1.815 (0.747-4.410)(0.189)	-
High risk CAs	4.243 (1.605-11.218)**(0.004)	4.268 (1.547-11.771)*(0.005)
Response after HDM/AuHSCT		
VGPR	ref	-
CR	1.190 (0.431-3.291)(0.737)	-
sCR	1.091 (0.344-3.455)(0.883)	-
Total recovery of immunoparesis after HDM/AuSCT	0.522 (0.208-1.310)(0.166)	-

*. Statistical significance, p<0.05; **. p<0.01

Abbreviations: Alb, albumin; ALP, alkaline phosphatase; Ca, calcium; CAs, cytogenetic abnormalities; CI, confidence interval; CR, complete response; Cr, creatinine; CRP, C-reactive protein; DSS, Durie-Salmon staging; EMD, extramedullary disease; Hb, hemoglobin; HDM/AuHSCT, high dose melphalan followed by autologous hematopoietic stem cell transplantation; ISS, International Staging System; LDH, lactate dehydrogenase; MM, multiple myeloma; PC, plasma cells; Pla, platelet; ref, reference; RpSCT, response period for the HDM/AuHSCT ; sCR, stringent CR; suPD-L1, soluble PD-L1; ULN, upper limit of normal range; VGPR, very good partial response; WBC, white blood cells

Supplementary Table S3: Cox regression analysis among levels of suPD-L1 and salient clinical features at diagnosis associated with overall survival in the 49 patients with VGPR or better after HDM/AuHSCT

	Univariate analysis	Multivariate analysis
Item	Hazard Ratio (95% CI)(*P*-value)	
sPD-L1 level (ng/mL)		
<= 4.54	ref	Ref
> 4.54	13.796 (1.658-114.818)*(0.015)	9.181 (1.069-78.820)*(0.043)
Sex		
Male	ref	-
Female	3.230 (0.624-16.711) (0.162)	-
Age >= 60 years	1.688 (0.320-8.911)(0.538)	-
Hb >= 10gm/dL	0.225 (0.027-1.868)(0.167)	-
WBC >= 4000/uL	0.571 (0.107-3.034)(0.510)	-
Pla >= 150k/uL	0.906 (0.172-4.763)(0.907)	-
LDH > ULN	7.453 (1.636-33.959)**(0.009)	-
Alb >= 3.5 gm/dL	0.951 (0.182-4.972)(0.952)	-
ALP > ULN	1.177 (0.260-5.321)(0.833)	-
ISS III vs ISS I/II	1.083 (0.209-5.603)(0.925)	-
DSS IIIa/b vs DSS I/II	1.647 (0.365-7.429)(0.516)	-
EMD	2.046 (0.389-10.750)(0.398)	-
Light chain type	31.060 (0.015-62299.104)(0.376)	-
IgG type	2.021 (0.391-10.441)(0.401)	-
PC > 50%	9.477 (1.110-80.955)*(0.040)	-
High risk CAs	18.759 (2.183-161.225)**(0.008)	10.406 (1.186-91.330)*(0.035)
Response after HDM/AuHSCT		
VGPR	ref	-
CR	0.268 (0.030-2.405)(0.240)	-
sCR	0.774 (0.140-4.263)(0.768)	-
Total recovery of immunoparesis after HDM/AuSCT	0.811 (0.181-3.640)(0.785)	-

*. Statistical significance, p<0.05; **. p<0.01

Abbreviations: Alb, albumin; ALP, alkaline phosphatase; Ca, calcium; CAs, cytogenetic abnormalities; CI, confidence interval; CR, complete response; Cr, creatinine; CRP, C-reactive protein; DSS, Durie-Salmon staging; EMD, extramedullary disease; Hb, hemoglobin; HDM/AuHSCT, high dose melphalan followed by autologous hematopoietic stem cell transplantation; ISS, International Staging System; LDH, lactate dehydrogenase; MM, multiple myeloma; PC, plasma cells; Pla, platelet; ref, reference; RpSCT, response period for the HDM/AuHSCT ; sCR, stringent CR; suPD-L1, soluble PD-L1; ULN, upper limit of normal range; VGPR, very good partial response; WBC, white blood cells

Supplementary Table S4: Correlation between immunological parameters and suPD-L1 levels

Patients	Absolute counts (k/μL)*		Correlation coefficient with suPD-L1			
			Pearson's	P-value	Spearman's	P-value
All (N=61)						
	Neutrophils	2.5±1.4	0.036	0.781	0.144	0.267
	Lymphocytes	2.0±1.1	0.064	0.623	0.000	0.999
	Monocytes	0.4±0.3	0.018	0.892	0.038	0.771
	N/L ratio	1.0±0.6	0.033	0.803	-0.123	0.344
>=VGPR (N=49)						
	Neutrophils	2.6±1.4	-0.025	0.866	0.097	0.505
	Lymphocytes	2.0±1.0	0.036	0.804	-0.061	0.675
	Monocytes	0.4±0.3	-0.006	0.970	-0.003	0.986
	N/L ratio	0.9±0.5	0.116	0.426	-0.096	0.514

*. Mean±SD
N/L, Neutrophil/Lymphocyte

Supplementary Table S5: Comparison between blood counts and recovery of immunoparesis in the high and normal-to-low groups among the 49 patients with VGPR or better after HDM/AuHSCT

Item	Group of suPD-L1 (N)		*P*-value
	H (17)	NL (32)	
	Mean±SD		
ANC (k/uL)	2.4±0.8	2.7±1.7	0.569
ALC (k/uL)	2.1±1.4	1.9±0.8	0.542
AMC (k/uL)	0.3±0.1	0.4±0.3	0.176
Density of $CD8^+$ CTLs (%)	9.2±9.2	6.3±3.7	0.150
Total recovery of non-M Igs [N (%)]	9 (53)	15 (47)	0.763

Abbreviations: suPD-L1, soluble PD-L1; H, high; NL, normal-to-low; ANC, absolute neutrophil count; ALC, absolute lymphocyte count; AMC, absolute monocyte count; CTLs, cytotoxic T lymphocytes; HDM/AuHSCT, high dose melphalan followed by autologous hematopoietic stem cell transplantation; non-M Igs, non myeloma immunoglobulins

Supplementary Table S6: Comparison between blood counts, recovery of immunoparesis, levels of suPD-L1 among different CD8$^+$ CTLs density scores

		CD8$^+$ CTLs			
Density Score		0	1	2	
Density (%)		0 to <= 10	> 10 to <= 20	> 20	
N		19	22	15	*P*-value
Absolute counts (k/µL)*					
	Neutrophil	2.5±1.0	2.2±0.8	3.1±2.2	0.180
	Lymphocyte	2.1±1.0	1.8±0.8	2.5±1.5	0.147
	Monocyte	0.5±0.4	0.3±0.1	0.4±0.2	0.156
N/L ratio*		0.9±0.3	0.9±0.5	1.2±1.1	0.246
suPD-L1 (ng/mL)*		3.7±1.4	4.7±2.7	3.5±0.9	0.145
Total recovery of immunoparesis (N, %)		(9, 47)	(11, 50)	(7, 47)	1.000

*. Mean±SD
CTLs, cytotoxic lymphocytes; N/L, Neutrophil/Lymphocyte; suPD-L1, soluble PD-L1
